# Derivation and performance of an end-of-life practice score aimed at interpreting worldwide treatment-limiting decisions in the critically ill

**DOI:** 10.1186/s13054-022-03971-9

**Published:** 2022-04-13

**Authors:** Spyros D. Mentzelopoulos, Su Chen, Joseph L. Nates, Jacqueline M. Kruser, Christiane Hartog, Andrej Michalsen, Nikolaos Efstathiou, Gavin M. Joynt, Suzana Lobo, Alexander Avidan, Charles L. Sprung, the End-of-life Practice Score Study Group, Wesley Ely, Erwin J. O. Kompanje, Mervyn Mer, Charles Feldman, Victoria Metaxa, Myrick C. Shinall, John Myburgh, Charikleia S. Vrettou

**Affiliations:** 1 First Department of Intensive Care Medicine, National and Kapodistrian University of Athens Medical School, Evaggelismos General Hospital, 45-47 Ipsilandou Street, 10675 Athens, Greece; 2 D2, K Lab, Department of Electrical and Computer Engineering, Rice University, Houston, TX USA; 3 Critical Care Department, The University of Texas MD Anderson Cancer Center, Houston, TX USA; 4 Division of Allergy, Pulmonary, and Critical Care Medicine, The University of Wisconsin School of Medicine and Public Health, Madison, USA; 5 Department of Anesthesiology and Intensive Care Medicine, Charité University Medicine Berlin, Berlin, Germany; 6 Klinik Bavaria, Kreischa, Germany; 7 Department of Anesthesiology, Critical Care, Emergency Medicine, and Pain Therapy, Konstanz Hospital, Konstanz, Germany; 8 School of Nursing, Institute of Clinical Sciences, College of Medical and Dental Sciences, University of Birmingham, Birmingham, UK; 9 Department of Anaesthesia and Intensive Care, The Chinese University of Hong Kong, Shatin, Hong Kong SAR China; 10 Critical Care Division – Faculty of Medicine São José do Rio Preto, São Paulo, Brazil; 11 Department of Anesthesiology, Critical Care and Pain Medicine, Hadassah Medical Organization and Faculty of Medicine, Hebrew University of Jerusalem, Jerusalem, Israel

**Keywords:** End-of-life practice score, ROC analysis, Medical ethics, End-of-life care, Palliative care, Life-sustaining therapy, Intensive care unit

## Abstract

**Background:**

Limitations of life-sustaining interventions in intensive care units (ICUs) exhibit substantial changes over time, and large, contemporary variation across world regions. We sought to determine whether a weighted end-of-life practice score can explain a large, contemporary, worldwide variation in limitation decisions.

**Methods:**

The 2015–2016 (Ethicus-2) vs. 1999–2000 (Ethicus-1) comparison study was a two-period, prospective observational study assessing the frequency of limitation decisions in 4952 patients from 22 European ICUs. The worldwide Ethicus-2 study was a single-period prospective observational study assessing the frequency of limitation decisions in 12,200 patients from 199 ICUs situated in 8 world regions. Binary end-of-life practice variable data (1 = presence; 0 = absence) were collected post hoc (comparison study, 22/22 ICUs, *n* = 4592; worldwide study, 186/199 ICUs, *n* = 11,574) for family meetings, daily deliberation for appropriate level of care, end-of-life discussions during weekly meetings, written triggers for limitations, written ICU end-of-life guidelines and protocols, palliative care and ethics consultations, ICU-staff taking communication or bioethics courses, and national end-of-life guidelines and legislation. Regarding the comparison study, generalized estimating equations (GEE) analysis was used to determine associations between the 12 end-of-life practice variables and treatment limitations. The weighted end-of-life practice score was then calculated using GEE-derived coefficients of the end-of-life practice variables. Subsequently, the weighted end-of-life practice score was validated in GEE analysis using the worldwide study dataset.

**Results:**

In comparison study GEE analyses, end-of-life discussions during weekly meetings [odds ratio (OR) 0.55, 95% confidence interval (CI) 0.30–0.99], end-of-life guidelines [OR 0.52, (0.31–0.87)] and protocols [OR 15.08, (3.88–58.59)], palliative care consultations [OR 2.63, (1.23–5.60)] and end-of-life legislation [OR 3.24, 1.60–6.55)] were significantly associated with limitation decisions (all *P* < 0.05). In worldwide GEE analyses, the weighted end-of-life practice score was significantly associated with limitation decisions [OR 1.12 (1.03–1.22); *P* = 0.008].

**Conclusions:**

Comparison study-derived, weighted end-of-life practice score partly explained the worldwide study’s variation in treatment limitations. The most important components of the weighted end-of-life practice score were ICU end-of-life protocols, palliative care consultations, and country end-of-life legislation.

**Supplementary Information:**

The online version contains supplementary material available at 10.1186/s13054-022-03971-9.

## Background

End-of-life care is an integral component in the delivery of critical care [1]. Epidemiological data indicate that 15–30% of patients admitted to intensive care units (ICUs) around the world die [2], while 10–12% undergo limitations of life-sustaining treatments [3–5]. Such treatments prolong life without reversing the underlying medical condition; examples include cardiopulmonary resuscitation (CPR), mechanical ventilation and renal replacement therapy [6].

In the past three decades, several studies have focused on the investigation of patients’, families’, physicians’ and nurses’ attitudes or practices regarding life support at the end-of-life [7–10]. The main concerns were symptom control, patient and family satisfaction, adequate communication and management of conflicts between individuals involved in end-of-life decision-making. Few studies have focused on the formal organizational or system-level support and the existing infrastructure of individual ICUs to assist health care staff to perform end-of-life care at a high standard [11].

The novel end-of-life practice score (EPS) is a 12-component score developed through expert consensus and review of existing literature. The EPS was designed to measure the end-of-life care infrastructure and organization of an ICU. It was first developed to interpret the increases in treatment limitations over time in 22 ICUs situated across Northern, Central and Southern Europe [4]. This was the principal finding of a two-part longitudinal study of ICU end-of-life care delivery, termed ″comparison study″ from now on. This comparison study had two data collection periods, 16 years apart, in the context of the Ethicus-1 study (1999–2000) and the Ethicus-2 study (2015–2016) [3–5]. Exploratory logistic regression analysis revealed a significant association between the EPS and time-dependent changes in the frequency of treatment limitation decisions [4]. However, the relative contribution of each one of the 12 binary end-of-life practice variables in explaining temporal changes in the frequency of limitation decisions remained unclear.

Using data from the comparison study’s European cohort [4], the current study aimed to first identify specific aspects of end-of-life practice with possibly strong, clinically relevant associations with the comparison study’s time-dependent variations in limitation decisions in European ICUs [4]. The second aim was to develop an EPS with appropriately weighted components. Such weighted EPS might aid in interpreting the recently reported global variation in treatment limitation frequency across 199 ICUs from 8 world regions (worldwide Ethicus-2 study) [5], and potentially have general application in future similar studies. Adequately interpreting this contemporary global variation might help improve end-of-life practice worldwide.

## Methods

This study includes data analyses from two previously approved and published studies [4, 5]. Therefore, there was no requirement for ethical approval.

### Comparison study summary description

Participating ICUs were in Northern (4 countries), Central (4 countries), and Southern (6 countries) Europe. Center-level and patient-level data were collected prospectively. Data on 4592 patients who died or had a limitation of life-sustaining interventions (2807 and 1785 from the Ethicus-1 and Ethicus-2 studies, respectively) were available [4]. Comparison study and worldwide Ethicus-2 study data forms and collection methodologies were identical [5].

The primary outcome was application of any limitation in life-prolonging therapy (withholding, or withdrawing, or active shortening of the dying process [4]). Patients were categorized into 5 prospectively defined and mutually exclusive end-of-life categories: withholding of life-sustaining therapy, withdrawing of life-sustaining therapy, active shortening of the dying process, failed CPR and brain death [4].

### Original (unweighted) EPS development

In the comparison study, 12 end-of-life practice variables (i.e., EPS subcomponents) were collected post hoc from 22 participating ICUs [4]. A simple questionnaire with two possible answers (i.e., no = “absence” or yes = “presence”) for each practice variable was administered electronically. These variables reflect key aspects of ICU end-of-life practice [4, 5] and include (1) routine family meetings [12–14], (2) daily deliberation for appropriate level of care [12], (3) end-of-life discussions during family meetings [12], (4) written triggers for treatment limitations [15, 16], (5–6) written end-of-life guidelines [17] and protocols [15], (7) palliative care consultations [14, 18], (8) ethics consultations [12, 14], (9–10) staff taking communication or bioethics courses [12–14, 18] and (11–12) country end-of-life guidelines or legislation [12, 17, 18]. Variables were graded by 0 or 1 according to their reported absence or presence, respectively. The sum of these grades was the “original” EPS, which ranged within 0–12. Thus, the higher the EPS the more end-of-life practices were concurrently present. Definitions of practice variables and the EPS are presented in Table 1. The same post hoc collection of binary end-of-life practice data was performed for the Ethicus-2 worldwide study [5].

**Table 1:** Definitions of subcomponent variables of end-of-life practice score and derivation of its weighted/rescaled form

EOL practice variable	Definition
Routine family meetings	Regular (i.e., on admission and at least twice a week) scheduled conferences of at least one member of an ICU patient’s family and at least one member of the treating team aimed at (a) determining/clarifying the patient’s health status, and comorbidities, (b) patient values, preferences, and goals concerning treatment options; and (c) conveying honest, accurate, and evidence-based information about patient clinical status and current/updated prognosis
Daily deliberation for appropriate level of care	Routine daily discussions among members of the ICU treating team aimed at confirming that medical/surgical interventions administered to a patient are not disproportionate and/or do not contradict his/her preferences
EOL discussions during family meetings	Conferences (on admission, and followed up at least as appropriate/feasible) of at least one member of an ICU patient’s family and at least one member of the treating team aimed at determining and/or revising/adjusting EOL treatment goals according to the evolution of the patient’s clinical course and (particularly changes) of prognosis, and “previously clarified” EOL values/preferences. This variable focuses on a specific type of family meetings’ content aimed at achieving consistency between patient wishes and provided EOL care
Written ICU triggers for limitations	A set of written, pre-specified medical and/or bioethical criteria for limiting LSTs in the ICU. Examples of such criteria may include: family request, presence of a pertinent living will that has to be respected, irreversible condition, un-survivable injury, severe brain injury with poor prognosis (e.g., minimally conscious state), high Sequential Organ Dysfunction Assessment Score plus]poor response to acute illness treatment, multiple organ failure (≥ 3 organs), non-beneficial therapy, and terminal illness
Written ICU EOL guidelines	Written ICU recommendations (e.g., shared decision-making, or obligation to inform the family about poor patient response to treatment, and/or lack of expected benefit from available and/or ongoing LSTs), with a written expectation to be followed for EOL decision-making and application of EOL decisions
Written ICU EOL (symptom management) protocols	A written set of ICU recommendations and standards aimed at preventing any kind of patient distress (e.g., pain, dyspnea, delirium) during the application of LST limitation decisions on withholding and/or withdrawing of LSTs); written ICU EOL protocols may be based on recent, pertinent recommendations on how to perform withdrawing of LSTs
Palliative care consultations	Consultations and/or liaison with specialists from the hospital’s (specifically designated) palliative care service, focused on the treatment of symptoms (e.g., dyspnea, pain, or delirium), rather than the treatment of any underlying disease processes. Psychosocial and spiritual needs may also be attended to in patients who do not require sedation and are able to communicate. Such consultations may take place whenever LST limitation is considered, in the context of communication of available treatment options to the patient/family. An exception to the former requirement pertains to the presence of an intensivist with palliative care expertise in the ICU treating team
Ethics consultations	Consultations and/or liaison with a specialist from the hospital’s (specifically designated) clinical ethics committee, focused on addressing of any ensuing ethical dilemmas and/or challenges, including disagreements (that cannot otherwise be resolved) between surrogate decision-makers, between the patient/family and the ICU treating team, health care professionals or others
Communication courses	Lessons focused at developing or improving the capability of (1) expressing oneself clearly, honestly, and accurately (about available treatment options), and also in a way that is readily understood by the patient/family; and (2) providing psychological support, and showing empathy to the patient/family
Bioethics courses	Lessons focused on improving the knowledge, understanding of the widely accepted four Principles of Bioethics, and/or the capability of effectively addressing ethical dilemmas and challenges of routine clinical practice
Country EOL guidelines	Written recommendations by national medical societies, or statutory governing bodies, for EOL decision-making and EOL practices (e.g., symptom control and/or procedure for withdrawal of mechanical ventilation) in the ICU
Country EOL legislation	A set of laws aimed at addressing commonly ensuing ethical issues as part of routine clinical practice (e.g., Should advance directives always be followed? Are withholding or withdrawing of LSTs, or active shortening of the dying process legally allowed?, etc.)
EOL practice score	The sum of binary (i.e., 0 or 1) grading of the 12 EOL practice variables according to their absence (= 0) or presence (= 1); score range: 0–12
Weighted EOL practice score	Sum of products of EOL practice variable grades and GEE coefficients derived from the GEE analysis of the comparison study data (see also “Methods”); sum actual range: − 2.574 to 5.706
Weighted EOL practice score rescaled to a 0 to 12 range^a^	Weighted/rescaled EOL practice score = [12/(5.706 + 2.574)]*(“actual” weighted EPS + 2.574)

*ICU* intensive care unit, *EOL* end-of-life, *LST* life-sustaining treatment, *GEE* generalized estimating equations

^**a**^This transformation was undertaken, in order to simplify/facilitate the interpretation of the weighted EPS odds ratio determined in the GEE analyses of the worldwide study data

### Derivation of the weighted EPS

**Fig. 1:**
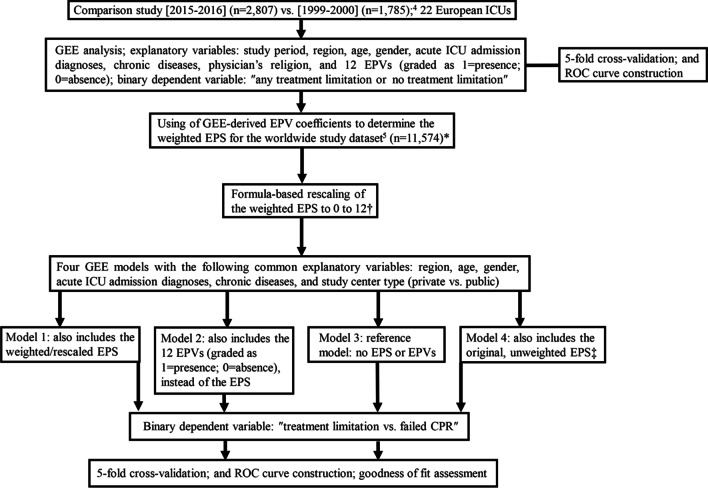
Flowchart of the employed analytic methodology. ICU, intensive care unit; GEE, generalized estimating equations; EPV, end-of-life practice variable; *ROC* receiver operating characteristic, *EPS* end-of-life practice score, *CPR* cardiopulmonary resuscitation. *The weighted EPS was determined by first multiplying the comparison study’s [4] GEE-derived EPV coefficients by the 0 or 1 response grades of the 12 EPVs from the worldwide dataset [5], and then by summing up the aforementioned products. ^†^The EPS rescaling formula is presented in Table 1. ^‡^The original, unweighted EPS was calculated as the sum of the 0 or 1 response grades of the 12 EPVs from the worldwide dataset [5]; author consensus definitions of the EPVs are provided in Table 1

The comparison study showed a substantial increase in treatment limitations’ frequency over time and a decrease in the frequency of death without limitation [4]. This was considered as a time-dependent improvement in end-of-life practices [19]. To determine the relative importance of each end-of-life practice variable as explanatory variable, generalized estimating equations (GEE) analysis with robust standard errors and an exchangeable working correlation structure accounting for the factor center [5] was applied to the entire comparison study population [4]. Additional explanatory variables included study period (i.e., 2015–2016 vs. 1999–2000), region (i.e., Northern, Central and Southern Europe), age, gender, acute ICU admission diagnoses, chronic diseases and physician religion. Type of model was set at “binary logistic.” The binary dependent variable was patients with “any treatment limitation or no treatment limitation” (Fig. 1). For these patient-level analyses, it was assumed that a specific, ICU-level grading of an end-of-life practice variable should correspond to all patients originating from that ICU. For example, if an ICU contributed 100 patients and the site principal investigator responded positively to “end-of-life discussions during weekly meetings”-meaning that this was a typical ICU-level characteristic-then “end-of-life discussions” were assumed to have occurred for all the 100 participants of that ICU [4].

Comparison study GEE model was cross-validated using the fivefold validation technique [20]. More specifically, the entire study dataset was randomly split into five, equally-sized groups, i.e., the fivefolds, with one of the folds (20% of the data) serving as the validation group and the remaining four folds (80% of the data) serving as the training group for constructing probabilistic models. The model was fit on the training group, and its coefficient estimates were used to predict treatment limitation probability in the validation group. This process was followed five times in total; each time, a different fold was used as validation group [20, 21].

Agreement (calibration) between predicted and observed treatment limitations in the validation group was assessed by constructing a receiver operating characteristic (ROC) curve based on the entire dataset and calculating the “area under the curve” (AUC).

### Weighted EPS rescaling and validation

Patient-level GEE analysis accounting for center on the worldwide Ethicus-2 dataset (*n* = 11,574) included a weighted EPS and the following explanatory variables: world region (i.e., Africa, Latin America, North America, Asia, Australia/New Zealand and Northern, Central and Southern Europe), age, gender, acute ICU admission diagnoses, chronic diseases, and center-type (i.e., private vs. public) (worldwide model 1; Fig. 1). The worldwide dataset did not include brain-dead patients, and the dependent variable remained ″limitation yes/no″ [5], reflecting treatment limitation vs. failed CPR. Weighted EPS was calculated by multiplying the 0 or 1 end-of-life practice variable response grades by the GEE coefficients determined in the comparison study data analysis and summing up the resulting 12 end-of-life practice variable-specific products (Table 1). Consequently, the weighted EPS was derived according to both the presence and relative importance of its subcomponents. Subsequently, EPS’s values were linearly transformed (i.e., rescaled) to its original 0–12 range [4] (Table 1).

Three additional GEE models were fit on the worldwide study data, namely a recently reported worldwide model 2 [5] and 2 additional models, i.e., worldwide models 3 and 4. Worldwide model 2 differed from worldwide model 1 in including the 12 end-of-life practice variables as separate explanatory variables instead of the EPS [5] (Fig. 1). Worldwide model 3 (reference model) included all the explanatory variables of models 1 and 2, besides the EPS or the end-of-life practice variables (Fig. 1). Worldwide model 4 included the variables of worldwide model 3 plus the original, unweighted EPS version (i.e., the simple sum of the 1/0 grades of the end-of-life practice variables [4, 5]) (Fig. 1). Weighted EPS validation was the primary aim of the fitting of worldwide model 1. The purpose of the additional fitting of worldwide models 2–4 was to comparatively determine any potential weighted EPS-associated improvement in GEE model performance. Analyses were designed by SDM, SC and JN.

All models were subjected to fivefold cross-validation. Furthermore, for all worldwide models, ROC construction and corresponding AUC determinations were used to assess agreement between predicted and observed treatment limitations in the validation groups. Finally, goodness of fit was compared between worldwide models 1, 2, and 4 vs. reference model 3 using the “analysis of variance (ANOVA)” function in R (Fig. 1).

EPS and failed CPR worldwide study data were compared among regions by Kruskal Wallis test and Pearson chi square test, respectively. All analyses were conducted with R (version 4.0.2). GEE analysis was conducted with R package geepack and Figures were produced using R package pROC [22]. Figure 1 is a summary illustration of the above-described analytic methodology. Additional methodological details are presented in Additional file 1.

## Results

### GEE model for weighted EPS derivation

Data from 4592 patients were included in the comparison study’s GEE model. Patient characteristics have been reported elsewhere [4] and are presented in Additional file 1: Table S1.

**Fig. 2:**
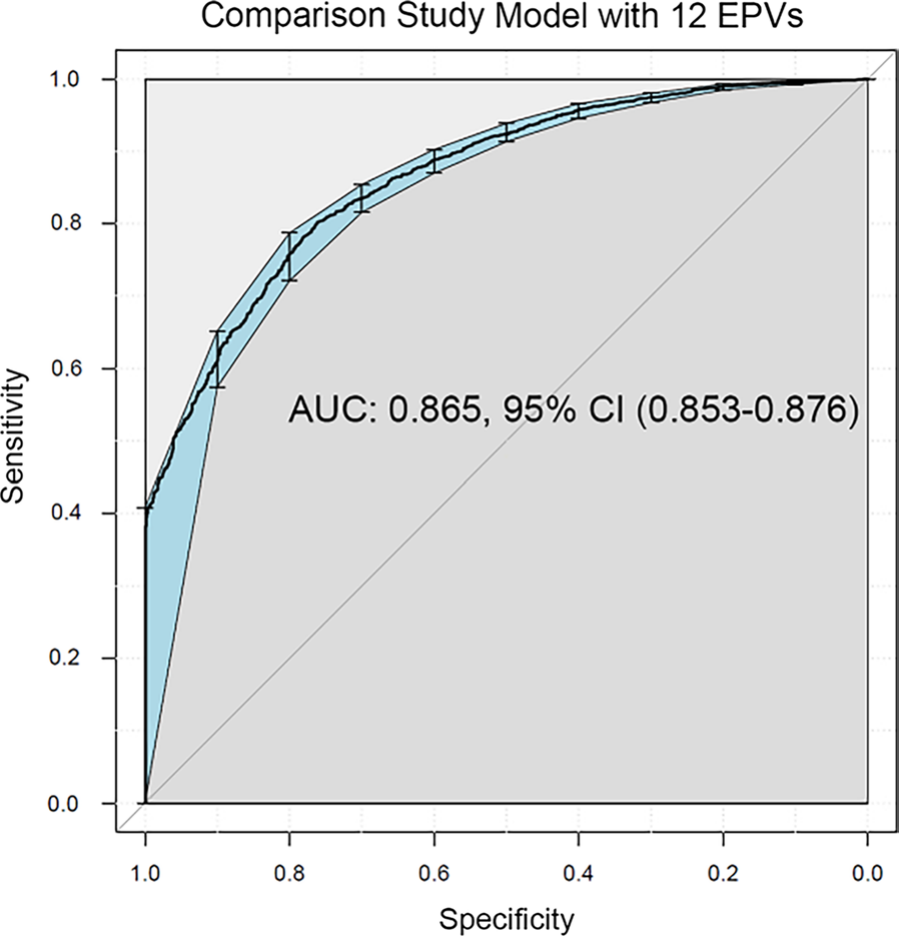
Receiver operating characteristic curve based on the comparison study’s [4] generalized estimating equations model

Table 2 displays comparison study GEE results (Ethicus-2 vs. Ethicus-1), which reconfirm that the 2015–2016 Ethicus-2 cohort was strongly associated with treatment limitation [odds ratio (OR): 36.3, (95% confidence interval): (9.1–144.5)]; patient age, physician religion, and acute diagnoses/chronic diseases were also associated with limitation decisions. Among end-of-life practice variables, end-of-life discussions during weekly meetings [OR 0.55, (0.30–0.99)], written ICU end-of-life guidelines [OR 0.52, (0.31–0.87)], written ICU end-of-life protocols [OR 15.08, (3.88–58.59)], palliative care consultations [OR 2.63, (1.23–5.60], and national end-of-life legislation [OR 3.24, (1.60–6.55)] were significantly associated with limitation decisions. The AUC of the comparison study model was 0.865 after applying fivefold cross-validation (Fig. 2).

**Table 2:** Comparison study general estimating equations model for “any treatment limitation or no treatment limitation”

	Estimate	OR	95% CI		*P* value
			Lower	Upper	
Ethicus 2 study (2015–2016) vs. Ethicus 1 study (1999–2000)	3.59	36.29	9.12	144.47	< 0.001
Region					
Central Europe vs. Northern Europe	− 0.20	0.82	0.42	1.59	0.56
Southern Europe vs. Northern Europe	− 1.13	0.32	0.15	0.68	0.003
Age	0.03	1.02	1.02	1.03	< 0.001
Sex, female vs. male	− 0.02	0.98	0.89	1.07	0.62
Physician religion					
Catholic vs. none	0.64	1.89	1.19	3.00	0.007
Jewish vs. none	1.04	2.83	1.48	5.41	0.002
Greek orthodox vs. none	0.55	1.74	0.97	3.10	0.06
Protestant vs. none	0.87	2.39	1.42	4.04	0.001
Unknown vs. none	− 6.03	0.002	0.001	0.010	< 0.001
Other vs. none	0.26	1.30	0.75	2.25	0.35
Islam vs. none	0.41	1.51	0.63	3.60	0.35
Acute diagnoses					
Surgery vs. neurologic	− 0.13	0.88	0.62	1.23	0.45
Respiratory vs. neurologic	0.49	1.64	1.16	2.32	0.006
Cardiovascular vs. neurologic	− 0.19	0.83	0.62	1.11	0.22
Gastrointestinal vs. neurologic	0.68	1.98	1.40	2.81	< 0.001
Metabolic vs. neurologic	0.70	2.01	1.00	4.06	0.0502
Hematologic vs. neurologic	0.48	1.62	0.71	3.74	0.26
Trauma vs. neurologic	− 0.32	0.73	0.51	1.05	0.09
Sepsis vs. neurologic	0.65	1.92	1.25	2.94	0.003
Other vs. neurologic	0.51	1.66	1.00	2.74	0.048
Chronic diseases					
Cardiovascular diseases vs. none	0.56	1.75	1.40	2.18	< 0.001
Neurological-cognitive diseases–muscular vs. none	0.74	2.09	1.25	3.48	0.005
Chest diseases vs. none	0.91	2.48	1.80	3.40	< 0.001
Kidney and urinary system diseases vs. none	0.38	1.47	0.88	2.44	0.14
Digestive system vs. none	1.49	4.46	2.72	7.29	< 0.001
Immunologic system vs. none	0.93	2.53	1.49	4.30	0.001
General history vs. none	0.43	1.54	1.14	2.07	0.005
Cancer vs. none	1.17	3.23	2.15	4.83	< 0.001
Unknown vs. none	1.30	3.67	1.56	8.65	0.003
End-of-life practice variables					
Routine ICU family meetings: yes vs. no	− 0.03	0.97	0.52	1.79	0.91
Daily deliberation for appropriate level of ICU care: yes vs. no	0.57	1.77	0.96	3.28	0.07
End-of-life (EOL) discussions during weekly (family) meetings: yes vs. no	− 0.61	0.55	0.30	0.99	0.047
Written triggers for limitations: yes vs. no	-0.14	0.87	0.41	1.86	0.72
Written ICU EOL guidelines: yes vs. no	− 0.65	0.52	0.31	0.87	0.013
Written ICU EOL protocols: yes vs. no	2.71	15.08	3.88	58.59	< 0.001
Palliative care consultations: yes vs. no	0.97	2.63	1.23	5.60	0.012
Ethics consultations: yes vs. no	− 0.96	0.38	0.14	1.07	0.07
ICU staff taking communication courses: yes vs. no	0.13	1.14	0.52	2.51	0.74
ICU staff taking bioethics courses: yes vs. no	− 0.19	0.83	0.19	3.57	0.80
Country EOL guidelines: yes vs. no	0.14	1.16	0.53	2.50	0.72
Country EOL legislation: yes vs. no	1.17	3.24	1.60	6.55	0.001
Intercept	− 1.54	0.21	0.08	0.59	0.003

Patient data originate from the entire comparison study population (*n* = 4592) (4)

*CI* confidence interval, *OR* odds ratio. Collinearity assessment: variance inflation, 1.03–4.29; condition index, 30.75

### GEE model for weighted EPS validation

In the worldwide study cohort, EPS/end-of-life practice variable data were available from 186/199 participating ICUs (93.5%), corresponding to 11,574 patients who died or had a treatment limitation [5]. Baseline characteristics of worldwide study participants are presented in Additional file 1: Table S2. Regional and overall original and weighted/rescaled EPS values and frequency distribution of failed CPR are presented in Table 3.

**Table 3:** Regional end-of-life practice score and frequency of failed cardiopulmonary resuscitation

Region	No. of centers	No. of patients	Original EPS median (IQR) ^a^	W/R EPS median (IQR)^a^	No. (%) of failed CPR^b^
Africa	2	160	2 (2–7)	3.67 (3.56–3.67)	106 (66.3)
Latin America	9	501	6 (3–7)	4.70 (4.70–7.47)	154 (30.7)
North America	9	910	9 (9–12)	8.76 (8.24–8.76)	78 (8.6)
Asia	28	1690	7 (4–7)	5.56 (3.96–7.03)	253 (15.0)
Australia/New Zealand	9	513	8 (7–8)	7.80 (5.73–9.81)	23 (4.5)
Central Europe	41	3494	7 (6–9)	6.47 (5.46–8.24)	402 (11.5)
Northern Europe	35	2055	7 (6–9)	5.72 (4.41–7.43)	70 (3.4)
Southern Europe	53	2251	6 (4–9)	5.51 (4.24–7.02)	553 (24.6)
Total	186	11,574	7 (6–9)	6.28 (4.50–8.22)	1,639 (14.2)

*EPS* end-of-life practice score, *IQR* interquartile range, *CPR* cardiopulmonary resuscitation, *W/R* weighted/rescaled

^a^Monte Carlo significance level of Kruskal Wallis test among the eight world regions, *P* < 0.001

^b^Significance level of “overall” Pearson chi square test among the eight world regions, *P* < 0.001

Worldwide GEE models 1, 2, and 3 and 4 are presented in Table 4, and Additional file 1: Tables S3, S4 and S5, respectively. As also elsewhere reported [5], region, age, acute diagnoses/chronic diseases and center type were associated with limitation decisions in all GEE models. In worldwide model 1, weighted/rescaled EPS was an independent predictor of treatment limitation [OR 1.12, (1.03–1.22)] (Table 4: EPS data highlighted in bold), i.e., for each 1-point increment in weighted/rescaled EPS, treatment limitation probability increased by 12%.

**Table 4:** Worldwide general estimating equations model 1 for ″treatment limitation vs. failed cardiopulmonary resuscitation.”

	Estimate	OR	95% CI		*P* value
			Lower	Upper	
Region					
America Latin vs. Africa	1.90	6.66	0.81	54.94	0.08
America Northern vs. Africa	2.58	13.20	1.47	118.28	0.02
Asia vs. Africa	2.57	13.10	1.73	98.89	0.013
Australia/New Zealand vs. Africa	3.31	27.35	3.32	225.03	0.002
Europe Central vs. Africa	2.20	9.02	1.20	67.81	0.03
Europe Northern vs. Africa	3.80	44.83	5.89	341.23	< 0.001
Europe Southern vs. Africa	2.05	7.79	1.05	58.08	0.045
Age	0.01	1.01	1.01	1.02	< 0.001
Sex, female vs. male	0.05	1.05	0.95	1.16	0.37
Acute diagnoses					
Surgery vs. neurologic	− 0.53	0.59	0.48	0.72	< 0.001
Respiratory vs. neurologic	− 0.54	0.58	0.49	0.70	< 0.001
Cardiovascular vs. neurologic	− 1.01	0.36	0.29	0.45	< 0.001
Gastrointestinal vs. neurologic	− 0.45	0.64	0.50	0.81	< 0.001
Metabolic vs. neurologic	− 0.52	0.60	0.43	0.83	0.002
Hematologic vs. neurologic	− 0.70	0.50	0.36	0.69	< 0.001
Trauma vs. neurologic	− 1.11	0.33	0.22	0.49	< 0.001
Sepsis vs. neurologic	− 0.61	0.54	0.45	0.67	< 0.001
Other vs. neurologic	− 0.99	0.37	0.25	0.55	< 0.001
Chronic diseases					
Cardiovascular diseases vs. none	0.13	1.14	0.95	1.36	0.17
Neurological-cognitive diseases–muscular vs. none	0.57	1.77	1.38	2.28	< 0.001
Chest vs. none	0.38	1.46	1.16	1.84	0.001
Kidney vs. none	0.14	1.15	0.88	1.50	0.31
Digestive system vs. none	0.47	1.60	1.22	2.09	0.001
Immunologic system vs. none	0.33	1.39	0.95	2.05	0.09
General history vs. none	0.24	1.28	1.02	1.60	0.04
Cancer vs. none	0.53	1.70	1.33	2.17	< 0.001
Unknown vs. none	− 0.26	0.77	0.56	1.07	0.12
Center type (private vs. public)	− 0.57	0.57	0.33	0.98	0.04
**Weighted and rescaled end-of-life practice score**	**0.12**	**1.12**	**1.03**	**1.22**	**0.008**
Intercept	− 1.94	0.14	0.02	1.11	0.06

Patient data originate from the entire worldwide study population (*n* = 11,574) [5]. The comparison-study [4] derived, weighted and rescaled end-of-life practice score is included as explanatory variable (see also Methods)

CI, confidence interval; OR, odds ratio. Collinearity assessment: variance inflation, 1.01–1.18; condition index, 18.32. The results on the variable of interest, i.e. the end-of-life practice score, are highlighted in bold

**Fig. 3:**
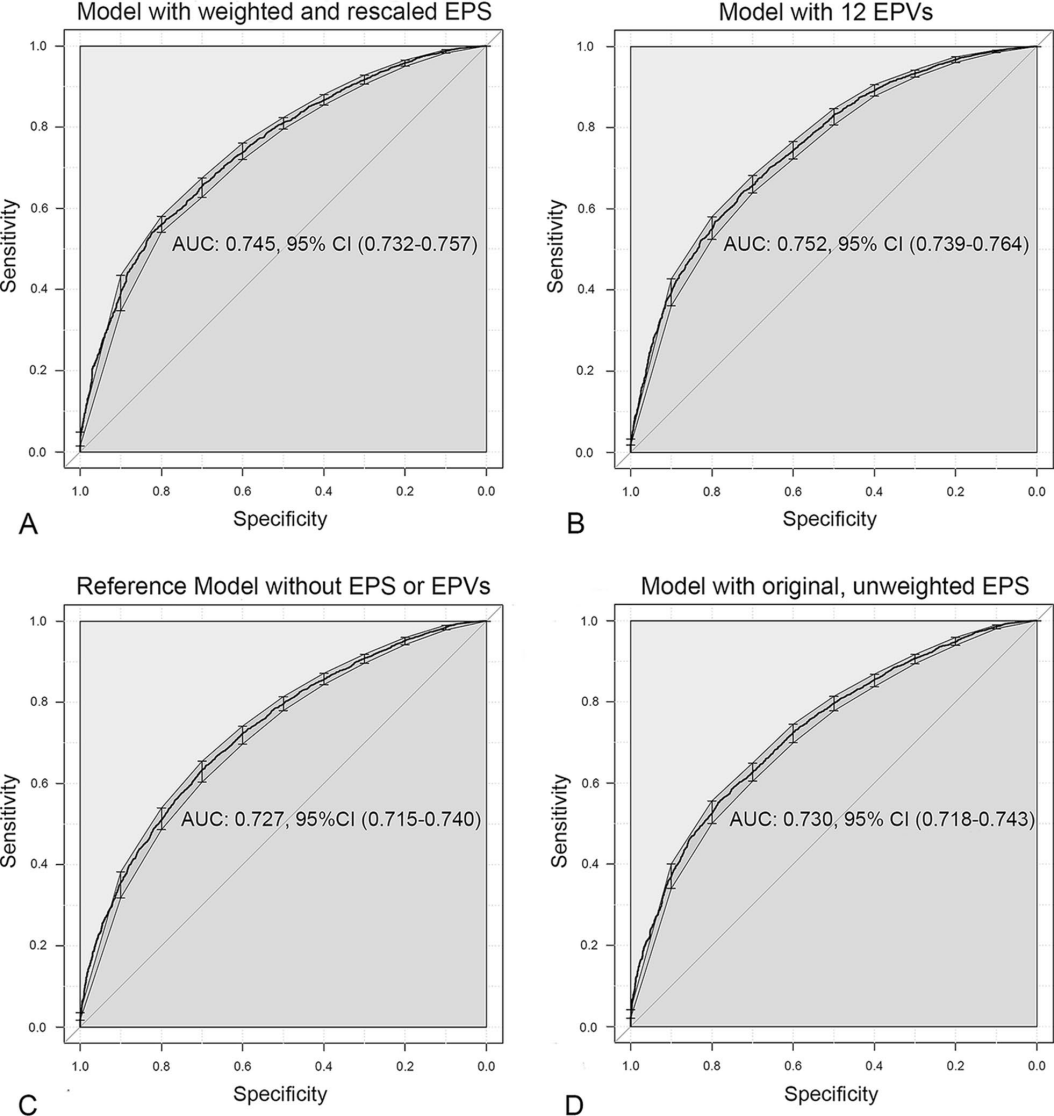
ROC curves of the 4 generalized estimating equations models of the worldwide study [5]. *ROC* receiver operating characteristic, *EPS* end-of-life practice score, *EPV* end-of-life practice variable, *AUC* area under the curve, *CI* confidence interval. **A**: Model with weighted and rescaled EPS (worldwide model 1); **B**: Model with EPVs (worldwide model 2); **C**: Reference model without EPVs or EPS (worldwide model 3); **D**: Model with original, unweighted EPS (worldwide model 4)

The AUCs of worldwide models 1, 2, 3, and 4 were 0.745, 0.752, 0.727, and 0.730, respectively (Fig. 3). Between-model comparisons (by R’s ″ANOVA″) demonstrated that only the worldwide model 1 had significantly better goodness of fit vs. the reference model (*P* = 0.008). In contrast, the goodness of fit of worldwide models 2 and 4 was not significantly better when compared to the reference model (*P* = 0.056–0.23) (Fig. 3).

### Additional exploratory analyses

In the worldwide study population [5], presence of country end-of-life legislation and/or combined presence of end-of-life practice variables with significant ORs (see also above and Table 2) was associated with failed CPR frequencies of 8.4–9.4%, whereas upper-quartile weighted/rescaled EPS of ≥ 8.22 was associated with a failed CPR frequency of < 8% (Table 5). Region-level proportions of ″raw″ positive responses to the 12 end-of-life practice variables for both the comparison and worldwide studies [4, 5] are provided in Additional file 1: Table S6. In the comparison study [4], maximal European regional increases over time in positive responses for end-of-life protocols, palliative care consultations, and end-of-life legislation amounted to 50%; the respective maximal differences in the positive responses of the worldwide study [5] varied within 68–100%.

**Table 5:** Worldwide study [5] frequency of failed cardiopulmonary resuscitation under specific conditions of end-of-life practice

	EOL legislation present	EOL legislation absent	Difference (95% CI)	*P* value
Failed CPR, no/total No., (%)	664/7070 (9.4)	975/4504 (21.6)	− 12.3 (− 13.6 to − 10.9)	< 0.001

	3 ″high-OR EPVs″ present	0–2 ″high-OR EPVs″ present	Difference (95% CI)	*P*- value
Failed CPR, no/total No., (%)	202/2398 (8.4)	1437/9176 (15.7)	− 7.2 (− 8.6 to − 5.9)	< 0.001

	5 ″significant″ EPVs present	0–4 ″significant″ EPVs present	Difference (95% CI)	*P *value
Failed CPR, no/total No., (%)	139/1548 (9.0)	1500/10,026 (15.0)	− 6.0 (− 7.6 to − 4.4)	< 0.001

	W/R EPS ≥ 8.22^a^	W/R EPS < 8.22	Difference (95% CI)	*P* value
Failed CPR, no/total No., (%)	231/2976 (7.8)	1408/8598 (16.4)	− 8.6 (− 9.9 to − 7.4)	< 0.001

EOL, end-of-life; CI, confidence interval; OR, odds ratio 3; EPV, end-of-life practice variable; ″high-OR EPVs″, written (departmental) EOL protocols, palliative care consultations, and national EOL legislation (see also Results and Table 2); 5 ″significant″ EPVs, the aforementioned 3 ″high-OR EPVs″ plus EOL discussions during weekly family meetings and written (departmental) guidelines (see also Results and Table 2); W/R, weighted/rescaled; EPS, end-of-life practice score

^a^Value represents upper-quartile W/R EPS (see also Results and Table 3)

Worldwide, country-level, weighted/rescaled EPS data are shown in Additional file 1: Figure S1.

## Discussion

A novel end-of-life practice score for ICUs, the EPS, weighted according to the strength of the associations of its subcomponents with limitation decisions, was derived from data obtained from a large ethical comparison study [4]. The weighted EPS was rescaled to 0–12, to align with the originally proposed score, and subsequently validated as explanatory variable for treatment limitation decisions using data from a larger worldwide study of end-of-life decision-making [5]. A high EPS was best achieved by ensuring combined presence of the three end-of-life practice variables with the highest coefficient estimates in the comparison study’s GEE analysis, namely the presence of end-of-life ICU protocols, palliative care consultations and national end-of-life legislation. Notably, in a hypothetical case of concurrent positive responses for these variables and negative responses for the remaining 9 variables, the weighted/rescaled EPS-value would amount to 10.76, which is quite close to its maximum value of 12.00. The results from the worldwide study’s data suggest that regions with a high weighted/rescaled EPS demonstrate increased frequency of life-support limitation, and a reduction in failed CPR. Indeed, an upper-quartile EPS was associated with failed CPR rates of < 8%.

In comparison study’s analyses, palliative care consultations had the third highest OR for predicting limitation decisions and the third highest coefficient in EPS weighting. Currently, palliative care is widely recognized as a key component of patient/family centered ICU care [13, 14, 19, 23–32]. Nevertheless, our data and other findings reveal that the presence of ICU-based palliative care may substantially vary across world regions (current study's range: 21–89%, corresponding to 68% variation), countries and hospitals [4, 5, 19, 33–36], and even among physicians working in the same ICU [37, 38], or according to daily ICU bed pressure [39].

Four systematic reviews suggested that consultative or integrative palliative care interventions may reduce ICU/hospital length of stay and cost, without increasing mortality [23–26]. Educational interventions aimed at ICU staff, and interventions comprising screening for palliative care referral, goals-of-care discussions and specialist palliative care involvement were associated with significant increases in limitation of life-sustaining treatments and CPR [26]. However, review findings were limited by study quality, heterogeneity of interventions/outcomes and uncertainty about generalizability [23–26].

Randomized trials of palliative care-led family meetings or complex, integrative interventions targeted at clinicians reported neutral and/or negative results, including worsening of post-traumatic stress disorder symptoms [26, 40, 41]. Conversely, randomized trials of multi-component interventions delivered by an interprofessional ICU team or of early-triggered palliative care consultations reported mainly positive results, including better clinician-family communication, more limitations in life-sustaining treatments and transitions to hospice care, shorter ICU stay or decreased ICU resource utilization and no significant effect on in-hospital mortality [42, 43]. Published data and current results support the need for further, evidence-based integration of well-designed and multifaceted palliative care interventions in standard ICU care [26]. Such interventions should result in timely provision of effective physical, psychological and spiritual comfort care by specifically trained/skilled ICU clinicians and/or palliative care specialists.

According to our findings, end-of-life protocols in the context of withdrawing or withholding life-sustaining measures should be considered a positive factor during the terminal period of provision of effective palliative care. End-of-life protocol application should be supported by a weighted shared decision that continuation of life-sustaining treatments would confer more harm than benefit to the individual patient [13, 14, 44–46]. End-of-life protocols should focus on the prevention/alleviation of any associated distressful patient symptoms (e.g., pain, dyspnea, or delirium) and minimization/prevention of any potential long-term psychological impact to family members (e.g., post-traumatic stress disorder, anxiety, depression, and complicated grief [16, 44, 45]). A preceding roundtable conference concluded that withdrawing of treatments such as mechanical ventilation should be tailored to individual patient needs [47]. A recent systematic review reported a worldwide variation and ambiguity of practices of withdrawal of mechanical ventilation [45]. Nevertheless, in countries from world regions with high (e.g., 100%) positive response rates for end-of-life protocols (e.g., USA), the quality of dying and death has also been rated high by families of decedent ICU patients [48]. Global variation in end-of-life protocol use is consistent with the worldwide study’s data, although the substantial temporal increase observed in the comparison study suggests that implementation remains dynamic and is evolving with time.

In several countries, the potential for exposure to legal risk may prevent ICU physicians from limiting invasive treatments (including CPR) in patients with poor prognosis [49]. End-of-life legislation is a well-established key factor not only for the prevention of disproportionate treatments [44], but also for the development of inter-professional decision-making and consensus building practices that take into account the patient’s values, goals and preferences, and ameliorate the moral distress associated with end-of-life decisions [46, 50]. Notably, legislative processes may be protracted, depending on cultural, religious, social, linguistic and political barriers, and the presence/intensity of lobbying/support by groups of stakeholders and members of regulatory bodies [51–53]. Furthermore, the implementation of in-force laws may still be limited due to lack of awareness, perceived ambiguity, or non-compliance by involved parties (e.g., healthcare professionals) [54, 55]. Nevertheless, the enactment of end-of-life legal frameworks, followed by the development of multifaceted end-of-life care programs/initiatives with electronic infrastructure (e.g., the Physician Orders for Life-Sustaining Treatments [56]) has been shown to substantially promote concordance between recorded patient wishes and administered end-of-life treatments and care [18, 57–59]. Our results confirm this relationship, as a strong association between end-of-life legislation and treatment limitation [5] was observed in all analyses.

Increased rates of limitation and consequent reduction in failed CPR may imply a higher quality of end-of-life care, strictly in the context of the concurrent presence of the above-discussed end-of-life practices and legal support. Nevertheless, decisions on life-support/CPR should still be individualized, taking into account patient prognosis and preferences. The Ethicus study protocol [3–5] did not include any collection of patient-level data on the fulfilment of criteria for withholding or withdrawing CPR [20].

End-of-life practice variable data were not uniformly consistent. Notably, in the comparison study analyses, end-of-life discussions and departmental end-of-life guidelines were negatively associated with treatment limitations. Recently reported limitations of clinician-family end-of-life conferences include insufficient information exchange about the patient’s values and preferences and deficient deliberation; this implies that the communication skills of clinicians need to be improved [18, 59]. Regarding guidelines per se, these may not effectively address problems of reaching consensus decisions, prognostication challenges, barriers in communication, patient palliative care needs, and physician-related variability in end-of-life decision-making [58–62]. Absence of association or negative associations between a number of the end-of-life practice variables and treatment limitation were noted, and appear to be counter-intuitive and difficult to explain. A possible explanation is that in the presence of end-of-life ICU protocols, palliative care consultations, and national end-of-life legislation, other local end-of-life practices may become somewhat redundant. Nevertheless, these local practices may also improve end-of-life care [12–18, 63] and should therefore be retained in the EPS, for further development in validation studies. Collectively, our results on the relative importance of 12 end-of-life practices highlight the need for further, high-quality research based improvement in interventions related to communication, ethics consultations, education, palliative care, and advance care planning or goals-of-care discussions [26]. The resulting progress in end-of-life practice might then be quantifiable by concurrent changes in the weighting of the corresponding EPS subcomponents.

Current results may also indicate the need for further evaluation and improvement of currently accepted end-of-life practices [5]. The establishment of EPS/end-of-life care Registries might facilitate the periodic (e.g., biyearly) determination of potential, time-dependent changes in the associations between the 12 end-of-life practice variables and treatment limitation. This should enable EPS reweighting (and subsequent prospective validation) according to the evolution of end-of-life care, thereby maintaining and/or enhancing its potential usefulness as a simple tool for continuous assessment and improvement of end-of-life care.

Strengths of the current analyses include using robust analytic methodology and large datasets to derive and validate the weighted/rescaled EPS. Limitations include the post hoc EPS data collection, which may have introduced recall and/or social desirability bias [4, 5]. Also, for the purpose of analyses, ICU-level responses for end-of-life practice variables were assumed to uniformly reflect individual patient-level practice; pertinent consequences could include (1) biased results on end-of-life practices with known, patient-level, qualitative variability (e.g., end-of-life discussions) [59], and (2) additional bias due to potential, physician-related variability in end-of-life practice [62, 64]. Nevertheless, our comparison study analysis was actually adjusted for physician religion, which partly explains end-of-life practice variation [3, 64]. Additional limitations comprise uncertainty about the validity and reliability of end-of-life practices derived by expert consensus, absence of prospective EPS validation and lack of data on patients not admitted to the ICU in the context of treatment limitation decisions in hospital wards [4, 5]. Potential perception and measurement bias cannot be excluded, since the presence of the variables was determined by subjective perception. Lastly, only physicians were asked and not nurses or patients/family members.

## Conclusions

A weighted/rescaled EPS developed on the basis of changes in limitation decisions over a 16-year period [4] partly explained the substantial variation in contemporary treatment limitation decisions observed in the worldwide study [5]. The most important weighted/rescaled EPS components were ICU end-of-life protocols, palliative care consultations, and country end-of-life legislation. ICUs wishing to improve quality of end-of-life care may consider introducing the palliative care and end-of-life protocols into their organizational structures. Furthermore, national lawmakers might consider establishing and/or improving country-specific end-of-life legislations and healthcare policies targeted at facilitating their implementation.

## Supplementary Information

**Additional file 1**. Online-only supplementary material containing supplemental Methods, supplemental Results, Tables S1, S2, S3, S4, S5 and S6, and Figure S1.

## Data Availability

The datasets used and/or analyzed during the current study are available from the corresponding author on reasonable request.

## Abbreviations

ICU: Intensive care unit
EPS: End-of-life practice score
CPR: Cardiopulmonary resuscitation
GEE: Generalized estimating equations
ROC: Receiver operating characteristic curve
AUC: Area under curve
ANOVA: Analysis of variance
OR: Odds ratio

## References

[1] Cook D, Rocker G (2014). Dying with dignity in the intensive care unit. N Engl J Med.

[2] Vincent JL, Marshall JC, Namendys-Silva SA, François B, Martin-Loeches I, Lipman J, et al; ICON investigators. Assessment of the worldwide burden of critical illness: the intensive care over nations (ICON) audit. Lancet Respir Med. 2014;2:380–6. 10.1016/S2213-2600(14)70061-X 24740011

[3] Sprung CL, Cohen SL, Sjokvist P, Baras M, Bulow HH, Hovilehto S, et al; Ethicus Study Group. End-of-life practices in European intensive care units: the Ethicus Study. JAMA. 2003;290:790–7. 10.1001/jama.290.6.790 12915432

[4] Sprung CL, Ricou B, Hartog CS, Maia P, Mentzelopoulos SD, Weiss M, et al. Changes in end-of-life practices in European intensive care units from 1999 to 2016. JAMA. 2019;322:1692–1704. Erratum in: JAMA. 2019;322:1718. 10.1001/jama.2019.14608 PMC6777263 31577037

[5] Avidan A, Sprung CL, Schefold JC, Ricou B, Hartog CS, Nates JL, et al; ETHICUS-2 Study Group. Variations in end-of-life practices in intensive care units worldwide (Ethicus-2): a prospective observational study. Lancet Respir Med. 2021: S2213-2600(21)00261-7. 10.1016/S2213-2600(21)00261-7 34364537

[6] Ko DN, Blinderman CD, Cherny N, Fallon M, Kaasa S, Portenoy RK, Currow DC (2015). Withholding and withdrawing life-sustaining treatment (including artificial nutrition and hydration). Oxford textbook of palliative medicine.

[7] Sjökvist P, Cook D, Berggren L, Guyatt G (1998). A cross-cultural comparison of attitudes towards life support limitation in Sweden and Canada. Clin Intensive Care.

[8] Prendergast TJ, Claessens MT, Luce JM (1998). A national survey of end-of-life care for critically ill patients. Am J Respir Crit Care Med.

[9] Vincent JL (1999). Forgoing life support in western European intensive care units: the results of an ethical questionnaire. Crit Care Med.

[10] Sprung CL, Carmel S, Sjokvist P, Baras M, Cohen SL, Maia P, et al; ETHICATT Study Group. Attitudes of European physicians, nurses, patients, and families regarding end-of-life decisions: the ETHICATT study. Intensive Care Med. 2007;33:104–10. 10.1007/s00134-006-0405-1 17066284

[11] Van den Bulcke B, Piers R, Jensen HI, Malmgren J, Metaxa V, Reyners AK (2018). Ethical decision-making climate in the ICU: theoretical framework and validation of a self-assessment tool. BMJ Qual Saf.

[12] Sprung CL, Truog RD, Curtis JR, Joynt GM, Baras M, Michalsen A (2014). Seeking worldwide professional consensus on the principles of end-of-life care for the critically ill. The Consensus for Worldwide End-of-Life Practice for Patients in Intensive Care Units (WELPICUS) study. Am J Respir Crit Care Med..

[13] Kon AA, Davidson JE, Morrison W, Danis M, White DB; American College of Critical Care Medicine; American Thoracic Society. Shared decision making in ICUs: an American College of Critical Care Medicine and American Thoracic Society Policy Statement. Crit Care Med. 2016;44:188–201. 10.1097/CCM.0000000000001396 PMC4788386 26509317

[14] Davidson JE, Aslakson RA, Long AC, Puntillo KA, Kross EK, Hart J (2017). Guidelines for family-centered care in the neonatal, pediatric, and adult ICU. Crit Care Med.

[15] Downar J, Delaney JW, Hawryluck L, Kenny L (2016). Guidelines for the withdrawal of life-sustaining measures. Intensive Care Med.

[16] Joynt GM, Lipman J, Hartog C, Guidet B, Paruk F, Feldman C (2015). The Durban World Congress Ethics Round Table IV: health care professional end-of-life decision making. J Crit Care.

[17] Guide on the decision-making process regarding medical treatment in end-of-life situations. Committee on Bioethics (DH-BIO) of the Council of Europe, 2014. https://www.coe.int/t/dg3/healthbioethic/conferences_and_symposia/Guide%20FDV%20E.pdf. Accessed 1/9/2022.

[18] Mentzelopoulos SD, Couper K, Voorde PV, Druwé P, Blom M, Perkins GD (2021). European Resuscitation Council Guidelines 2021: ethics of resuscitation and end of life decisions. Resuscitation.

[19] Cox CE, Hua M, Casarett D (2019). A measured dose of optimism for the evolution of ICU-based palliative care. JAMA.

[20] Fushiki T (2011). Estimation of prediction error by using K-fold cross-validation. Stat Comput.

[21] Yadav S, Shukla S. Analysis of k-fold cross-validation over hold-out validation on colossal datasets for quality classification. In: 2016 IEEE 6th international conference on advanced computing (IACC); 2016; p. 78–83.

[22] Halekoh U, Højsgaard S, Yan J (2006). The R package geepack for generalized estimating equations. J Stat Softw.

[23] Aslakson R, Cheng J, Vollenweider D, Galusca D, Smith TJ, Pronovost PJ (2014). Evidence-based palliative care in the intensive care unit: a systematic review of interventions. J Palliat Med.

[24] Khandelwal N, Kross EK, Engelberg RA, Coe NB, Long AC, Curtis JR (2015). Estimating the effect of palliative care interventions and advance care planning on ICU utilization: a systematic review. Crit Care Med.

[25] Kyeremanteng K, Gagnon LP, Thavorn K, Heyland D, D'Egidio G (2018). The impact of palliative care consultation in the ICU on length of stay: a systematic review and cost evaluation. J Intensive Care Med.

[26] Metaxa V, Anagnostou D, Vlachos S, Arulkumaran N, Bensemmane S, van Dusseldorp I (2021). Palliative care interventions in intensive care unit patients. Intensive Care Med.

[27] Hope AA, Enilari OM, Chuang E, Nair R, Gong MN (2021). Prehospital frailty and screening criteria for palliative care services in critically ill older adults: an observational cohort study. J Palliat Med.

[28] Hua MS, Li G, Blinderman CD, Wunsch H (2014). Estimates of the need for palliative care consultation across United States intensive care units using a trigger-based model. Am J Respir Crit Care Med.

[29] Nelson JE, Curtis JR, Mulkerin C, Campbell M, Lustbader DR, Mosenthal AC, et al; Improving Palliative Care in the ICU (IPAL-ICU) Project Advisory Board. Choosing and using screening criteria for palliative care consultation in the ICU: a report from the Improving Palliative Care in the ICU (IPAL-ICU) Advisory Board. Crit Care Med. 2013;41:2318–27. 10.1097/CCM.0b013e31828cf12c 23939349

[30] Ford DW (2014). Palliative care consultation needs in United States intensive care units. Another workforce shortage?. Am J Respir Crit Care Med..

[31] Lamas DJ, Owens RL, Bernacki RE, Block SD (2014). Palliative care: a core competency for intensive care unit doctors. Am J Respir Crit Care Med.

[32] Truog RD, Campbell ML, Curtis JR, Haas CE, Luce JM, Rubenfeld GD, et al; American Academy of Critical Care Medicine. Recommendations for end-of-life care in the intensive care unit: a consensus statement by the American College [corrected] of Critical Care Medicine. Crit Care Med. 2008;36:953–63. Erratum in: Crit Care Med. 2008;36:1699. 10.1097/CCM.0B013E3181659096 18431285

[33] Woitha K, Garralda E, Martin-Moreno JM, Clark D, Centeno C (2016). Ranking of palliative care development in the countries of the European Union. J Pain Symptom Manag.

[34] Pivodic L, Pardon K, Van den Block L, Van Casteren V, Miccinesi G, Donker GA, et al; EURO IMPACT. Palliative care service use in four European countries: a cross-national retrospective study via representative networks of general practitioners. PLoS ONE. 2013;8:e84440. 10.1371/journal.pone.0084440 PMC3875565 24386381

[35] Yamaguchi T, Kuriya M, Morita T, Agar M, Choi YS, Goh C (2017). Palliative care development in the Asia-Pacific region: an international survey from the Asia Pacific Hospice Palliative Care Network (APHN). BMJ Support Palliat Care.

[36] Clark D, Wright M, Hunt J, Lynch T (2007). Hospice and palliative care development in Africa: a multi-method review of services and experiences. J Pain Symptom Manag.

[37] Garland A, Connors AF (2007). Physicians' influence over decisions to forego life support. J Palliat Med.

[38] Calle MC, Pareja SL, Villa MM, Román-Calderón JP, Lemos M, Navarro S (2020). Interactions between intensive care and palliative care are influenced by training, professionals' perceptions and institutional barriers. J Palliat Care.

[39] Hua M, Halpern SD, Gabler NB, Wunsch H (2016). Effect of ICU strain on timing of limitations in life-sustaining therapy and on death. Intensive Care Med.

[40] Curtis JR, Nielsen EL, Treece PD, Downey L, Dotolo D, Shannon SE (2011). Effect of a quality-improvement intervention on end-of-life care in the intensive care unit: a randomized trial. Am J Respir Crit Care Med.

[41] Carson SS, Cox CE, Wallenstein S, Hanson LC, Danis M, Tulsky JA (2016). Effect of palliative care-led meetings for families of patients with chronic critical illness: a randomized clinical trial. JAMA.

[42] White DB, Angus DC, Shields AM, Buddadhumaruk P, Pidro C, Paner C, et al; PARTNER Investigators. A randomized trial of a family-support intervention in intensive care units. N Engl J Med. 2018;378:2365–75. 10.1056/NEJMoa1802637 29791247

[43] Ma J, Chi S, Buettner B, Pollard K, Muir M, Kolekar C, Al-Hammadi N (2019). Early palliative care consultation in the medical ICU: a cluster randomized crossover trial. Crit Care Med.

[44] Mularski RA, Curtis JR, Billings JA, Burt R, Byock I, Fuhrman C (2006). Proposed quality measures for palliative care in the critically ill: a consensus from the Robert Wood Johnson Foundation Critical Care Workgroup. Crit Care Med.

[45] Efstathiou N, Vanderspank-Wright B, Vandyk A, Al-Janabi M, Daham Z, Sarti A (2020). Terminal withdrawal of mechanical ventilation in adult intensive care units: a systematic review and narrative synthesis of perceptions, experiences and practices. Palliat Med.

[46] Michalsen A, Long AC, DeKeyser GF, White DB, Jensen HI, Metaxa V (2019). Interprofessional shared decision-making in the ICU: a systematic review and recommendations from an expert panel. Crit Care Med.

[47] Paruk F, Kissoon N, Hartog CS, Feldman C, Hodgson ER, Lipman J (2014). The Durban World Congress Ethics Round Table Conference Report: III. Withdrawing mechanical ventilation–the approach should be individualized. J Crit Care..

[48] Gerritsen RT, Koopmans M, Hofhuis JG, Curtis JR, Jensen HI, Zijlstra JG (2017). Comparing quality of dying and death perceived by family members and nurses for patients dying in US and Dutch ICUs. Chest.

[49] Phua J, Joynt GM, Nishimura M, Deng Y, Myatra SN, Chan YH, et al; ACME Study Investigators and the Asian Critical Care Clinical Trials Group. Withholding and withdrawal of life-sustaining treatments in intensive care units in Asia. JAMA Intern Med. 2015;175:363–71. Erratum in: JAMA Intern Med. 2015;175:1248. 10.1001/jamainternmed.2014.7386 25581712

[50] Collier J, Kelsberg G, Safranek S (2018). Clinical Inquiries: How well do POLST forms assure that patients get the end-of-life care they requested?. J Fam Pract.

[51] Andorno R, Biller-Andorno N, Brauer S (2009). Advance health care directives: towards a coordinated European policy?. Eur J Health Law.

[52] Sulmasy DP (2018). Italy's new advance directive law: when in Rome…. JAMA Intern Med.

[53] Ciliberti R, Gorini I, Gazzaniga V, De Stefano F, Gulino M (2018). The Italian law on informed consent and advance directives: new rules of conduct for the autonomy of doctors and patients in end-of-life care. J Crit Care.

[54] Evans N, Bausewein C, Meñaca A, Andrew EV, Higginson IJ, Harding R, et al; project PRISMA. A critical review of advance directives in Germany: attitudes, use and healthcare professionals' compliance. Patient Educ Couns. 2012;87:277–88. 10.1016/j.pec.2011.10.004 22115975

[55] Bolcato M, Feola A, Sanavio M, Amadasi A, Crenna S, Landi G (2020). The state of knowledge of young Italian medicolegal doctors on the law of provisions for informed consent and advance treatment directives: a multi-centric survey two years after the enactment of Law 219 of 2017. Acta Biomed..

[56] Tolle SW, Teno JM (2017). Lessons from Oregon in embracing complexity in end-of-life care. N Engl J Med.

[57] Petrova M, Riley J, Abel J, Barclay S (2018). Crash course in EPaCCS (Electronic Palliative Care Coordination Systems): 8 years of successes and failures in patient data sharing to learn from. BMJ Support Palliat Care.

[58] Park SY, Lee B, Seon JY, Oh IH (2021). A national study of life-sustaining treatments in South Korea: what factors affect decision-making?. Cancer Res Treat.

[59] Scheunemann LP, Ernecoff NC, Buddadhumaruk P, Carson SS, Hough CL, Curtis JR (2019). Clinician-family communication about patients' values and preferences in intensive care units. JAMA Intern Med.

[60] Sengupta J, Chatterjee SC (2017). Dying in intensive care units of India: commentaries on policies and position papers on palliative and end-of-life care. J Crit Care.

[61] Lilley EJ, Williams KJ, Schneider EB, Hammouda K, Salim A, Haider AH (2016). Intensity of treatment, end-of-life care, and mortality for older patients with severe traumatic brain injury. J Trauma Acute Care Surg.

[62] Wilkinson DJ, Truog RD (2013). The luck of the draw: physician-related variability in end-of-life decision-making in intensive care. Intensive Care Med.

[63] Schneiderman LJ, Gilmer T, Teetzel HD, Dugan DO, Blustein J, Cranford R (2003). Effect of ethics consultations on nonbeneficial life-sustaining treatments in the intensive care setting: a randomized controlled trial. JAMA.

[64] Löfmark R, Nilstun T, Cartwright C, Fischer S, van der Heide A, Mortier F, et al; EURELD Consortium. Physicians' experiences with end-of-life decision-making: survey in 6 European countries and Australia. BMC Med. 2008;6:4. 10.1186/1741-7015-6-4 PMC2277432 18269735

