# Supplementary material for: Derivation and performance of an end-of-life practice score aimed at interpreting worldwide treatment-limiting decisions in the critically ill
Source: Crit Care. 2022 Apr 13;26:106. doi: 10.1186/s13054-022-03971-9 (PMC9009016; doi:10.1186/s13054-022-03971-9)
Supplement: Supplementary file 1 — Additional file 1. Online-only supplementary material containing supplemental Methods, supplemental Results, Tables S1, S2, S3, S4, S5 and S6, and Figure S1. [file 13054_2022_3971_MOESM1_ESM.docx]

**ADDITIONAL FILE 1:**

**POST HOC DEVELOPMENT AND PERFORMANCE OF A WEIGHTED END-OF-LIFE PRACTICE SCORE AS EXPLANATORY VARIABLE OF VARIATION IN LIMITATION DECISIONS**

Spyros D. Mentzelopoulos, MD, Su Chen, PhD; Joseph L. Nates, et al; on behalf of the End-of-life Practice Score Study Group.

**CONTENTS**

**1] Supplemental Methods –** Page 1

**2] Supplemental Results –** Page 1

Tables S1 to S6 – Pages 2 to 10

Figure S1 – Page 11

References – Page 12

This additional file has been provided by the authors to give readers additional information about their work.

**Supplemental Methods**

The homogeneity of the comparison study’s end-of-life practice variable questionnaire [1] was assessed by the equivalent of the Kuder Richardson 20 coefficient [2], the Cronbach’s Alpha, for dichotomous data using ʺAlpha Modelsʺ in R version 4.0.2.

In sensitivity analyses, worldwide generalized estimating equations (GEE) analyses (see also Methods of the main paper) were repeated after excluding 1,711 European patients who were also included in comparison study’s GEE analysis [1, 3].

For all GEE models, multicollinearity was assessed by variance inflation and condition index. Goodness of fit was compared between worldwide total population and sensitivity models 1, 2, and 4 vs. reference model 3 using the ʺanalysis of variance (ANOVA)ʺ function in R. In comparing the fits of two GEE models, the ʺANOVAʺ function is equivalent to an asymptotic chi-square test employing the Wald statistic.

**Supplemental Results**

Cronbach’s alpha for ICU-level responses to the end-of-life practice variable questionnaire was 0.719. Additional study data and results are presented below in Tables S1 to S6 and Figure S1. Regarding multicollinearity, condition indices fluctuated within 15.68-30.75 (see footnotes of Tables 2 and 4 of the main paper, and of the below-provided Tables S3, S4 and S5), suggesting absence of any substantial problem(s) [4].

Results of sensitivity analyses were similar and are therefore not presented in detail; weighted/rescaled EPS odds ratio for treatment limitation was 1.12 (1.02-1.22) (P=0.016).

# **Table S1.** Study Population of the 22 1999 – 2000 (n = 2,807) and 2015 – 2016 (n = 1,785) European centers; these centers participated in both the comparison [1] and the worldwide [3] studies.

| **Characteristics** | **1999 - 2000** | | | **2015 – 2016 Difference (95% CI) 2015 -**  **2016 minus 1999 - 2000^a^** | |
| --- | --- | --- | --- | --- | --- |
| **Age, median (IQR)** | 67 (54-75) | | | 70 (59-79) 4.8 (3.8-5.8) | |
| **Sex, No. (%)** |  | | |  | |
| Male | 1719 (61.3) | | | 1079 (60.4) -0.8 (-3.7-2.1) | |
| Female | 1085 (38.7) | | | 706 (39.6) 0.9 (-2.0-3.8) | |
| **Patients by region, No (%)** | | | | | |
| Northern Europe | 587 (20.9) | | | 424 (23.8) 2.8 (0.4-5.3) | |
| Central Europe | 906 (32.3) | | | 893 (50.0) 17.8 (14.9-20.6) | |
| Southern Europe | 1314 (46.8) | | | 468 (26.2) -20.6 (-23.3- -17.8) | |
| **Physician religion, No. (%) of patients** | | | | | |
| Catholic | | 1066 (38.0) | 600 (33.6) | | -4.4 (-5.6- -3.1) |
| None | | 605 (21.6) | 259 (14.5) | | -7.0 (-8.1- -6.0) |
| Jewish | | 385 (13.7) | 79 (4.4) | | -9.3 (-10.0- -8.5) |
| Greek orthodox | | 348 (12.4) | 114 (6.4) | | -6.0 (-6.8- -5.2) |
| Protestant | | 295 (10.5) | 155 (8.9) | | -1.8 (-2.6- -1.0) |
| Other | | 49 (1.7) | 101 (5.7) | | 3.9 (3.4- 4.4) |
| Islam | | 35 (1.2) | 71 (4.0) | | 2.7 (2.3-3.2) |
| Unknown | | 24 (0.9) | 404 (22.6) | | 21.8 (20.9-22.6) |
| **ICU admission (acute) diagnoses, No (%)** | | | | | |
| Respiratory | 539 (19.2) | | | 431 (24.1) 4.9 (2.5-7.4) | |
| Cardiovascular | 478 (17.0) | | | 322 (18.0) 1.0 (-1.3-3.3) | |
| Neurologic | 472 (16.8) | | | 277 (15.5) -1.3 (-3.5-0.9) | |
| Surgery | 348 (12.4) | | | 339 (19.0) 6.6 (4.4-8.8) | |
| Gastrointestinal | 388 (13.8) | | | 98 (5.5) -8.3 (-10.0- -6.7) | |
| Sepsis | 248 (8.8) | | | 194 (10.9) 2.0 (0.2-3.8) | |
| Trauma | 196 (7.0) | | | 28 (1.6) -5.4 (-6.5- -4.3) | |
| Metabolic | 57 (2.0) | | | 44 (2.5) 0.4 (-0.5-1.3) | |
| Miscellaneous | 53 (1.9) | | | 38 (2.1) 0.2 (-0.6-1.1) | |
| Hematologic | 28 (1.0) | | | 14 (0.8) -0.2 (-0.8-0.3) | |

**Table S1.** (continued)

| **Characteristics 1999-2000 2015-2016 Difference (95% CI) 2015 - 2016**  **minus 1999 - 2000^a^** | | |
| --- | --- | --- |
| **Chronic diseases, No (%)** | | |
| Cardiovascular | 942 (33.6) | 758 (42.5) 8.9 (6.0-11.8) |
| None | 624 (22.2) | 140 (7.8) -14.4 (-16.4- -12.4) |
| Chest Diseases | 313 (11.2) | 180 (10.1) -1.1 (-2.9-0.8) |
| General History | 275 (9.8) | 175 (9.8) 0.0 (-1.8-1.8) |
| Cancer | 253 (9.0) | 179 (10.0) 1.0 (-0.7-1.8) |
| Neurological-Cognitive Diseases – Muscular | 135 (4.8) | 141 (7.9) 3.1 (1.6-4.6) |
| Digestive | 130 (4.6) | 99 (5.5) 0.9 (-0.4-2.2) |
| Kidney & Urinary System Diseases | 71 (2.5) | 64 (3.6) 1.1 (0.0-2.1) |
| Immunologic | 64 (2.3) | 34 (1.9) -0.4 (-1.2-0.5) |
| Missing data | 0 (0.0) | 15 (0.8) 0.8 (0.4-1.3) |

# CI, confidence interval; ICU, intensive care unit.

# ^a^, Regarding age, Difference (95% CI), represents mean difference (95% CI) ʺ2015-2016 minus 1999-2000 valuesʺ; regarding all other variables, Difference (95% CI) represents difference in percentages (95% CI); all differences were determined as 2015-2016 values minus 1999-2000 values. Adapted with permission from reference 1, which coincides with reference 4 of the main paper.

**Table S2.** Worldwide (WW) total [3] and sensitivity population characteristics.

|  | **Total WW study population with EPS/EPV data**  **N=11,574** | **Sensitivity WW study population**  **N=9,863** | **Difference (95% CI)**  **total minus minus sensitivity population^a^** |
| --- | --- | --- | --- |
| **Patients by region, No (%)** |  |  |  |
| Africa | 160 (1.4) | 160 (1.6) | -0.2 (-0.5-0.1) |
| America Latin | 501 (4.3) | 501 (5.1) | -0.8 (-1.4- -0.2) |
| America North | 910 (7.9) | 910 (9.2) | -1.3 (-2.1- -0.5 |
| Asia | 1690 (14.6) | 1690 (17.1) | -2.5 (-3.5- - 1.5) |
| Australia/New Zealand | 513 (4.4) | 513 (5.2) | -0.8 (-1.4- -0.2) |
| Europe Central | 3494 (30.2) | 2628 (26.6) | 3.6 (2.4-4.8) |
| Europe Northern | 2055 (17.9) | 1640 (16.6) | 1.3 (0.3-2.3) |
| Europe Southern | 2251 (19.4) | 1821 (18.5) | 0.9 (-0.2-2.0) |
| **Age, Median (IQR) (years)** | 70 (59-79) | 70 (58-79) | 0.2 (-0.9-0.9) |
| **Sex, No (%)** |  |  |  |
| Female | 6918 (59.8) | 5881 (59.6) | 0.2 (-1.2-1.5) |
| Male | 4656 (40.2) | 3982 (40.4) | -0.2 (-1.5-1.2) |
| **ICU admission (acute) diagnoses, No (%)** | | | |
| Respiratory | 3327 (28.7) | 2899 (29.4) | -0.6 (-1.9-0.6) |
| Cardiovascular | 1929 (16.7) | 1619 (16.4) | 0.3 (-0.7-1.2) |
| Surgery | 1864 (16.1) | 1536 (15.6) | 0.5 (-0.4-1.5) |
| Neurologic | 1538 (13.3) | 1303 (13.2) | 0.1 (-0.8-1.0) |
| Sepsis | 1316 (11.4) | 1122 (11.4) | 0.0 (-0.9-0.8) |
| Gastrointestinal | 735 (6.4) | 637 (6.5) | -0.1 (-0.8-0.5) |
| Metabolic | 359 (3.1) | 316 (3.2) | -0.1 (-0.6-0.4) |
| Miscellaneous | 192 (1.7) | 154 (1.6) | 0.1 (-0.2-0.4) |
| Trauma | 181 (1.6) | 158 (1.6) | 0.0 (-0.4-0.3) |
| Hematologic | 133 (1.1) | 119 (1.2) | -0.1 (-0.3-0.2) |
| **Chronic Diagnoses, No. (%)** |  |  |  |
| Cardiovascular Diseases | 4733 (40.9) | 4010 (40.7) | 0.2 (-1.1-1.6) |
| Chest Diseases | 1179 (10.2) | 1006 (10.2) | 0.0 (-0.8-0.8) |
| Cancer | 1170 (10.1) | 992 (10.1) | 0.1 (-0.8-0.9) |
| General History | 1087 (9.4) | 916 (9.3) | 0.1(-0.7-0.9) |
| None | 995 (8.6) | 875 (8.9) | -0.3 (-1.0-0.5) |
| Neurological-Cognitive Diseases–Muscular | 866 (7.5) | 727 (7.4) | 0.1(-0.6-0.8) |
| Digestive System | 579 (5.0) | 481 (4.9) | 0.1 (-0.5-0.7) |
| Kidney and Urinary System Diseases | 437 (3.8) | 374 (3.8) | 0.0 (-0.5-0.5) |
| Immunologic System | 282 (2.4) | 250 (2.5) | -0.1 (-0.5-0.3) |
| Unknown | 246 (2.1) | 232 (2.4) | -0.2 (-0.6-0.2) |
| **Center type - No. (%) of patients** |  |  |  |
| Private | 10439 (90.2) | 8810 (89.3) | 0.9 (0.1-1.7) |
| Public | 1135 (9.8) | 1053 (10.7) | -0.9 (-1.7- -0.1) |

# CI, confidence interval; ICU, intensive care unit.

^a^, Regarding age, Difference (95% CI), represents mean difference (95% CI) ʺtotal population minus sensitivity population valuesʺ; regarding all other variables, Difference (95% CI) represents difference in percentages (95% CI); all differences were determined as total population minus sensitivity population values. Adapted with permission from reference 3, which coincides with reference 5 of the main paper.

**Table S3.** Worldwide general estimating equations model 2 for ″treatment limitation vs. failed cardiopulmonary resuscitationʺ with the 12 end-of-life practice variables; patient data originate from the entire worldwide study population (n=11,574) [3].

| \| **Estimate OR 95% CI P-value**  **Lower Upper** \| \| --- \| | | | | | | | | | | | | | | | | | | | | | | | | |  |
| --- | --- | --- | --- | --- | --- | --- | --- | --- | --- | --- | --- | --- | --- | --- | --- | --- | --- | --- | --- | --- | --- | --- | --- | --- | --- | --- |
| **Region** | |  | | | | |  | | | | |  | |  | | | | |  | | | | | |  |
| America Latin vs. Africa | | | | 1.87 | | | | | | | 6.51 | | 0.87 | | 48.94 | | | | | 0.07 | |  |  |  |  |
| America Northern vs. Africa | | | | 2.05 | | | | | | | 7.79 | | 0.87 | | 69.30 | | | | | 0.07 | |  |  |  |  |
| Asia vs. Africa | | | | 2.44 | | | | | | | 11.49 | | 1.66 | | 79.58 | | | | | 0.013 | |  |  |  |  |
| Australia/New Zealand vs. Africa | | | | 2.98 | | | | | | | 19.75 | | 2.44 | | 159.60 | | | | | 0.005 | |  |  |  |  |
| Europe Central vs. Africa | | | | 1.82 | | | | | | | 6.18 | | 0.87 | | 43.87 | | | | | 0.07 | |  |  |  |  |
| Europe Northern vs. Africa | | | | 3.52 | | | | | | | 33.63 | | 4.61 | | 245.33 | | | | | 0.001 | |  |  |  |  |
| Europe Southern vs. Africa | | | | 1.79 | | | | | | | 5.99 | | 0.89 | | 40.60 | | | | | 0.07 | |  |  |  |  |
| **Age** | | | 0.01 | | | | | | 1.01 | | | | 1.02 | | | 1.03 | | | | | <0.001 | | |  |  |
| **Sex, Female vs. Male** | | | 0.05 | | | | | | 1.05 | | | | 0.95 | | | 1.16 | | | | | 0.35 | | |  |  |
| **Acute diagnoses** | | |  | | | | | |  | | | |  | | |  | | | | |  | | |  |  |
| Surgery vs. Neurologic | | | -0.54 | | | | | | 0.58 | | | | 0.47 | | | 0.72 | | | | | <0.001 | | |  |  |
| Respiratory vs. Neurologic | | | -0.54 | | | | | | 0.58 | | | | 0.49 | | | 0.69 | | | | | <0.001 | | |  |  |
| Cardiovascular vs. Neurologic | | | -1.03 | | | | | | 0.36 | | | | 0.29 | | | 0.44 | | | | | <0.001 | | |  |  |
| Gastrointestinal vs. Neurologic | | | -0.46 | | | | | | 0.63 | | | | 0.49 | | | 0.81 | | | | | <0.001 | | |  |  |
| Metabolic vs. Neurologic | | | -0.52 | | | | | | 0.59 | | | | 0.43 | | | 0.82 | | | | | 0.002 | | |  |  |
| Hematologic vs. Neurologic | | | -0.71 | | | | | | 0.49 | | | | 0.35 | | | 0.69 | | | | | <0.001 | | |  |  |
| Trauma vs. Neurologic | | | -1.13 | | | | | | 0.32 | | | | 0.22 | | | 0.48 | | | | | <0.001 | | |  |  |
| Sepsis vs. Neurologic | | | -0.62 | | | | | | 0.54 | | | | 0.44 | | | 0.66 | | | | | <0.001 | | |  |  |
| Other vs. Neurologic | | | -1.00 | | | | | | 0.37 | | | | 0.25 | | | 0.55 | | | | | <0.001 | | |  |  |
| **Chronic Diseases** | | |  | | | | | |  | | | |  | | |  | | | | |  | | |  |  |
| Cardiovascular Diseases vs. None | | | 0.12 | | | | | | 1.13 | | | | 0.94 | | | 1.36 | | | | | 0.20 | | |  |  |
| Neurological-Cognitive Diseases–Muscular vs. None | | | 0.58 | | | | | | 1.78 | | | | 1.38 | | | 2.30 | | | | | <0.001 | | |  |  |
| Chest vs. None | | | 0.38 | | | | | | 1.46 | | | | 1.15 | | | 1.84 | | | | | 0.002 | | |  |  |
| Kidney vs. None | | | 0.13 | | | | | | 1.13 | | | | 0.86 | | | 1.49 | | | | | 0.37 | | |  |  |
| Digestive System vs. None | | | 0.46 | | | | | | 1.59 | | | | 1.21 | | | 2.10 | | | | | 0.001 | | |  |  |
| immunologic System vs. None | | | 0.33 | | | | | | 1.39 | | | | 0.95 | | | 2.04 | | | | | 0.09 | | |  |  |
| General History vs. None | | | 0.24 | | | | | | 1.27 | | | | 1.01 | | | 1.60 | | | | | 0.04 | | |  |  |
| Cancer vs. None | | | 0.53 | | | | | | 1.70 | | | | 1.33 | | | 2.18 | | | | | <0.001 | | |  |  |
| Unknown vs. None | | | -0.27 | | | | | | 0.76 | | | | 0.54 | | | 1.07 | | | | | 0.11 | | |  |  |
| Center type (private vs. public) | | | -0.67 | | | | | | 0.51 | | | | 0.29 | | | 0.91 | | | | | 0.02 | | |  |  |
| **End-of-life practice variables** | | | |  | | |  | | | | |  | | | | |  | |  | | | | | |  |
| Routine ICU family meetings: Yes vs. No | | | | | -0.10 | | | | 0.90 | | | 0.50 | | | | 1.63 | | | 0.73 | | |  |  |  |  |
| Daily deliberation for appropriate level of ICU care: Yes vs. No | | | | | -0.12 | | | | 0.89 | | | 0.42 | | | | 1.87 | | | 0.76 | | |  |  |  |  |
| End-of-life (EOL) discussions during weekly (family) meetings: Yes vs. No | | | | | -0.11 | | | | 0.90 | | | 0.56 | | | | 1.43 | | | 0.65 | | |  |  |  |  |
| Written triggers for limitations: Yes vs. No | | | | | 0.01 | | | | 1.01 | | | 0.62 | | | | 1.63 | | | 0.98 | | |  |  |  |  |
| Written ICU EOL guidelines: Yes vs. No | | | | | -0.02 | | | | 0.98 | | | 0.59 | | | | 1.62 | | | 0.93 | | |  |  |  |  |
| Written ICU EOL protocols: Yes vs. No | | | | | 0.05 | | | | 1.05 | | | 0.63 | | | | 1.76 | | | 0.85 | | |  |  |  |  |
| Palliative care consultations: Yes vs. No | | | | | 0.51 | | | | 1.67 | | | 1.00 | | | | 2.79 | | | 0.05 | | |  |  |  |  |
| Ethics consultations: Yes vs. No | | | | | -0.01 | | | | 0.99 | | | 0.64 | | | | 1.53 | | | 0.97 | | |  |  |  |  |
| ICU Staff taking Communication Courses: Yes vs. No | | | | | 0.07 | | | | 1.07 | | | 0.67 | | | | 1.70 | | | 0.77 | | |  |  |  |  |
| ICU Staff taking Bioethics Courses: Yes vs. No | | | | | -0.21 | | | | 0.81 | | | 0.50 | | | | 1.31 | | | 0.39 | | |  |  |  |  |
| Country EOL Guidelines: Yes vs. No | | | | | -0.11 | | | | 0.90 | | | 0.54 | | | | 1.49 | | | 0.68 | | |  |  |  |  |
| **Table S3 (continued)** | | | | |  | | | |  | | |  | | | |  | | |  | | |  |  |  |  |
| \| **Estimate OR 95% CI P-value**  **Lower Upper** \| \| --- \| | | | | | | | | | | | | | | | | | | | | | | | | | |
| Country EOL Legislation: Yes vs. No | | | | | 0.75 | | | | 2.13 | | | 1.32 | | | | 3.41 | | | 0.002 | | |  |  |  |  |
| Intercept | | | | | -1.20 | | | | 0.30 | | | 0.04 | | | | 2.21 | | | 0.24 | | |  |  |  |  |

CI, confidence interval; OR, odds ratio. Reproduced with permission from reference 11, which coincides with reference 5 of the main paper.. Collinearity assessment: variance inflation, 1.01 – 1.73; condition index, 29.18.

**eTable S4.** Worldwide general estimating equations model 3 for ″treatment limitation vs. failed cardiopulmonary resuscitationʺ**,** not including the end-of-life practice score or the end-of-life practice variables (reference model); patient data originate from the entire worldwide study population (n=11,574) [3].

| \|  \| **Estimate OR 95% CI P-value**  **Lower Upper** \| \| --- \| --- \| | | | | | |
| --- | --- | --- | --- | --- | --- | --- | --- |
| **Region** |  |  |  |  |  |
| America Latin vs. Africa | 1.67 | 5.33 | 0.82 | 34.84 | 0.08 |
| America Northern vs. Africa | 2.88 | 17.75 | 2.49 | 126.61 | 0.004 |
| Asia vs. Africa | 2.52 | 12.39 | 2.10 | 72.95 | 0.005 |
| Australia/New Zealand vs. Africa | 3.59 | 36.33 | 5.83 | 226.56 | <0.001 |
| Europe Central vs. Africa | 2.36 | 10.56 | 1.81 | 61.61 | 0.009 |
| Europe Northern vs. Africa | 3.83 | 45.86 | 7.75 | 271.39 | <0.001 |
| Europe Southern vs. Africa | 1.98 | 7.27 | 1.26 | 41.78 | 0.03 |
| **Age** | 0.01 | 1.01 | 1.01 | 1.02 | <0.001 |
| **Sex, Female vs. Male** | 0.04 | 1.05 | 0.95 | 1.15 | 0.36 |
| **Acute diagnoses** |  |  |  |  |  |
| Surgery vs. Neurologic | -0.51 | 0.60 | 0.49 | 0.73 | <0.001 |
| Respiratory vs. Neurologic | -0.52 | 0.60 | 0.50 | 0.70 | <0.001 |
| Cardiovascular vs. Neurologic | -0.98 | 0.38 | 0.31 | 0.46 | <0.001 |
| Gastrointestinal vs. Neurologic | -0.44 | 0.65 | 0.51 | 0.81 | <0.001 |
| Metabolic vs. Neurologic | -0.50 | 0.61 | 0.45 | 0.83 | 0.002 |
| Hematologic vs. Neurologic | -0.67 | 0.51 | 0.37 | 0.70 | <0.001 |
| Trauma vs. Neurologic | -1.06 | 0.35 | 0.24 | 0.50 | <0.001 |
| Sepsis vs. Neurologic | -0.59 | 0.55 | 0.46 | 0.67 | <0.001 |
| Other vs. Neurologic | -0.95 | 0.39 | 0.26 | 0.57 | <0.001 |
| **Chronic Diseases** |  |  |  |  |  |
| Cardiovascular Diseases vs. None | 0.12 | 1.13 | 0.95 | 1.35 | 0.18 |
| Neurological-Cognitive Diseases–Muscular vs. None | 0.55 | 1.74 | 1.36 | 2.21 | <0.001 |
| Chest vs. None | 0.37 | 1.44 | 1.15 | 1.80 | 0.001 |
| Kidney vs. None | 0.13 | 1.14 | 0.88 | 1.47 | 0.32 |
| Digestive System vs. None | 0.45 | 1.56 | 1.21 | 2.03 | 0.001 |
| immunologic System vs. None | 0.31 | 1.36 | 0.94 | 1.97 | 0.11 |
| General History vs. None | 0.23 | 1.26 | 1.01 | 1.57 | 0.04 |
| Cancer vs. None | 0.51 | 1.66 | 1.31 | 2.10 | <0.001 |
| Unknown vs. None | -0.25 | 0.78 | 0.57 | 1.07 | 0.13 |
| **Center type** **(private vs. public)** | -0.43 | 0.65 | 0.39 | 1.08 | 0.10 |
| Intercept | -1.29 | 0.28 | 0.05 | 1.55 | 0.14 |

CI, confidence interval; OR, odds ratio. Collinearity assessment: variance inflation, 1.01 – 1.20; condition index, 15.68.

**Table S5.** Worldwide general estimating equations model 4 for ″treatment limitation vs. failed cardiopulmonary resuscitationʺ**,** including the original end-of-life practice score as sum of the 1 or 0 grades of the end-of-life practice variables; patient data originate from the entire worldwide study population (n=11,574) [3].

| \|  \| **Estimate OR 95% CI P-value**  **Lower Upper** \| \| --- \| --- \| | | | | | |
| --- | --- | --- | --- | --- | --- | --- | --- |
| **Region** |  |  |  |  |  |
| America Latin vs. Africa | 1.56 | 4.78 | 0.63 | 36.16 | 0.13 |
| America Northern vs. Africa | 2.60 | 13.47 | 1.56 | 116.05 | 0.018 |
| Asia vs. Africa | 2.43 | 11.41 | 1.66 | 78.38 | 0.013 |
| Australia/New Zealand vs. Africa | 3.41 | 30.13 | 4.08 | 222.83 | 0.001 |
| Europe Central vs. Africa | 2.20 | 9.07 | 1.32 | 62.43 | 0.03 |
| Europe Northern vs. Africa | 3.69 | 40.07 | 5.76 | 278.77 | <0.001 |
| Europe Southern vs. Africa | 1.90 | 6.66 | 0.99 | 44.87 | 0.051 |
| **Age** | 0.01 | 1.01 | 1.01 | 1.02 | <0.001 |
| **Sex, Female vs. Male** | 0.05 | 1.05 | 0.95 | 1.15 | 0.35 |
| **Acute diagnoses** |  |  |  |  |  |
| Surgery vs. Neurologic | -0.52 | 0.59 | 0.49 | 0.72 | <0.001 |
| Respiratory vs. Neurologic | -0.52 | 0.59 | 0.50 | 0.70 | <0.001 |
| Cardiovascular vs. Neurologic | -0.99 | 0.37 | 0.30 | 0.46 | <0.001 |
| Gastrointestinal vs. Neurologic | -0.44 | 0.64 | 0.51 | 0.81 | <0.001 |
| Metabolic vs. Neurologic | -0.50 | 0.60 | 0.44 | 0.83 | 0.002 |
| Hematologic vs. Neurologic | -0.68 | 0.51 | 0.37 | 0.70 | <0.001 |
| Trauma vs. Neurologic | -1.07 | 0.34 | 0.24 | 0.50 | <0.001 |
| Sepsis vs. Neurologic | -0.60 | 0.55 | 0.45 | 0.67 | <0.001 |
| Other vs. Neurologic | -0.96 | 0.38 | 0.26 | 0.56 | <0.001 |
| **Chronic Diseases** |  |  |  |  |  |
| Cardiovascular Diseases vs. None | 0.12 | 1.13 | 0.95 | 1.35 | 0.18 |
| Neurological-Cognitive Diseases–Muscular vs. None | 0.56 | 1.74 | 1.36 | 2.23 | <0.001 |
| Chest vs. None | 0.37 | 1.45 | 1.15 | 1.82 | 0.001 |
| Kidney vs. None | 0.14 | 1.15 | 0.88 | 1.49 | 0.30 |
| Digestive System vs. None | 0.45 | 1.57 | 1.21 | 2.05 | 0.001 |
| immunologic System vs. None | 0.31 | 1.37 | 0.94 | 2.00 | 0.10 |
| General History vs. None | 0.24 | 1.27 | 1.02 | 1.58 | 0.04 |
| Cancer vs. None | 0.51 | 1.67 | 1.32 | 2.12 | <0.001 |
| Unknown vs. None | -0.24 | 0.78 | 0.57 | 1.08 | 0.14 |
| **Center type** **(private vs. public)** | -0.41 | 0.67 | 0.40 | 1.11 | 0.12 |
| **Original, unweighted end-of-life practice score** | **0.05** | **1.05** | **0.97** | **1.14** | **0.23** |
| Intercept | -1.50 | 0.22 | 0.03 | 1.51 | 0.13 |

CI, confidence interval; OR, odds ratio. Collinearity assessment: variance inflation, 1.01 – 1.16; condition index, 18.10.

**Table S6.** Percentages of positive responses to the end-of-life practice variables in the comparison [1] and the worldwide [3] studies.

| **COMPARISON STUDY** | | | | | | | | | | | | | | |
| --- | --- | --- | --- | --- | --- | --- | --- | --- | --- | --- | --- | --- | --- | --- |
| Region | No. Countries / No. of ICUs | No. of Patients | Routine ICU family meetings | Daily deliberation for appropriate ICU care | EOL discussions  during  weekly family meetings | Written ICU triggers for limitations | Written ICU EOL guidelines | Written ICU EOL protocols | Palliative  Care  Consultations | Ethics consultations | ICU Staff taking Communication Courses | ICU Staff taking Bioethics Courses | Country EOL Guidelines | Country EOL Legislation |
| Europe C. 1999-2000 | 4/6 | 906 | 67 | 17 | 33 | 17 | 17 | 0 | 0 | 50 | 0 | 0 | 33 | 50 |
| Europe C.  2015-2016 | 4/6 | 893 | 83 | 100 | 67 | 33 | 33 | 17 | 50 | 50 | 50 | 17 | 50 | 50 |
| Europe N.  1999-2000 | 4/4 | 587 | 50 | 50 | 50 | 25 | 50 | 0 | 0 | 0 | 25 | 25 | 25 | 75 |
| Europe N.  2015-2016 | 4/4 | 424 | 50 | 50 | 75 | 50 | 75 | 50 | 25 | 0 | 100 | 50 | 100 | 75 |
| Europe S.  1999-2000 | 6/12 | 1314 | 75 | 58 | 50 | 17 | 17 | 8 | 8 | 0 | 17 | 8 | 0 | 0 |
| Europe S.  2015-2016 | 6/12 | 468 | 83 | 75 | 58 | 33 | 42 | 17 | 25 | 8 | 42 | 17 | 25 | 50 |
| **WORLDWIDE STUDY** | | | | | | | | | | | | | | |
| Region | No. Countries / No. of ICUs | No. of Patients | Routine ICU family meetings | Daily deliberation for appropriate level of ICU ICU care | EOL discussions  during  weekly family meetings | Written triggers for limitations | Written ICU EOL guidelines | Written ICU EOL protocols | Palliative  Care  Consultations | Ethics consultations | ICU Staff taking Communication Courses | ICU Staff taking Bioethics Courses | Country EOL Guidelines | Country EOL Legislation |
| Africa | 2/2 | 160 | 50 | 100 | 100 | 0 | 0 | 0 | 50 | 50 | 50 | 50 | 0 | 0 |
| America L. | 3/9 | 501 | 100 | 100 | 78 | 67 | 22 | 33 | 22 | 44 | 56 | 33 | 11 | 11 |
| America N. | 2/9 | 910 | 78 | 100 | 56 | 56 | 89 | 100 | 89 | 100 | 56 | 33 | 100 | 100 |
| Asia | 5/28 | 1690 | 61 | 86 | 43 | 64 | 43 | 43 | 32 | 36 | 50 | 25 | 61 | 29 |
| Australia/NZ | 2/9 | 513 | 100 | 89 | 78 | 56 | 33 | 56 | 78 | 33 | 89 | 22 | 78 | 78 |
| Europe C. | 7/41 | 3494 | 83 | 93 | 66 | 41 | 44 | 41 | 49 | 49 | 41 | 27 | 100 | 80 |
| Europe N. | 5/35 | 2055 | 91 | 74 | 69 | 40 | 74 | 37 | 49 | 29 | 66 | 23 | 89 | 54 |
| Europe S. | 7/53 | 2251 | 92 | 87 | 75 | 42 | 40 | 30 | 21 | 32 | 51 | 28 | 47 | 49 |

L., Latin; N., North/Northern; C., Central; S., Southern; NZ, New Zealand; ICU, intensive care unit; EOL, end-of-life.

**Figure S1.** Mean values (center of open dot symbols) and their 95% confidence intervals (bars) of the country-specific weighted and rescaled end-of-life practice score. Data originate from the entire worldwide study population (n=11,574) (3). Numbers above the dot symbols reflect country-specific percentages of treatment limitations. Numbers below the dot symbols are country-specific numbers of participating centers. Numbers just above the horizontal axis are numbers of study participants from each one of the 34 countries.

**References**

1. Sprung CL, Ricou B, Hartog CS, Maia P, Mentzelopoulos SD, Weiss M, et al. Changes in End-of-Life Practices in European Intensive Care Units From 1999 to 2016. JAMA. 2019;322:1692-1704. Erratum in: JAMA. 2019;322:1718.
2. Heale R, Twycross A. Validity and reliability in quantitative studies. Evid Based Nurs. 2015;18:66-67.
3. Avidan A, Sprung CL, Schefold JC, Ricou B, Hartog CS, Nates JL, et al; ETHICUS-2 Study Group. Variations in end-of-life practices in intensive care units worldwide (Ethicus-2): a prospective observational study. Lancet Respir Med. 2021: S2213-2600(21)00261-7.
4. Hair JF Jr, Black WC, Babin BJ, Anderson RE. Logistic regression: Regression with a binary independent variable. In: Hair JF Jr. Black WC, Babin BJ, Anderson RE, eds. Multivariable Data Analysis (7th ed). Edinburgh Gate, Harlow, Essex, UK: Pearson Education Ltd; 2014. p. 318-40.
